# DAMP-Inducing Adjuvant and PAMP Adjuvants Parallelly Enhance Protective Type-2 and Type-1 Immune Responses to Influenza Split Vaccination

**DOI:** 10.3389/fimmu.2018.02619

**Published:** 2018-11-20

**Authors:** Tomoya Hayashi, Masatoshi Momota, Etsushi Kuroda, Takato Kusakabe, Shingo Kobari, Kotaro Makisaka, Yoshitaka Ohno, Yusuke Suzuki, Fumika Nakagawa, Michelle S. J. Lee, Cevayir Coban, Risako Onodera, Taishi Higashi, Keiichi Motoyama, Ken J. Ishii, Hidetoshi Arima

**Affiliations:** ^1^Department of Physical Pharmaceutics, Graduate School of Pharmaceutical Sciences, Kumamoto University, Kumamoto, Japan; ^2^Laboratory of Adjuvant Innovation, Center for Vaccine and Adjuvant Research, National Institute of Biomedical Innovation, Health and Nutrition, Osaka, Japan; ^3^Laboratory of Vaccine Science, Immunology Frontier Research Center, Osaka University, Osaka, Japan; ^4^Program for Leading Graduate Schools “Health Life Science: Interdisciplinary and Global Oriented Program”, Kumamoto University, Kumamoto, Japan; ^5^Laboratory of Malaria Immunology, Immunology Frontier Research Center, Osaka University, Osaka, Japan; ^6^Building Regional Innovation Ecosystems, School of Pharmacy, Kumamoto University, Kumamoto, Japan

**Keywords:** 2-hydroxypropyl-β-cyclodextrin, K3 CpG-ODN, adjuvant, damage-associated molecular patterns, pathogen-associated molecular patterns, influenza split vaccine

## Abstract

Recently, it was reported that 2-hydroxypropyl-β-cyclodextrin (HP-β-CyD), a common pharmaceutical additive, can act as a vaccine adjuvant to enhance protective type-2 immunogenicity to co-administered seasonal influenza split vaccine by inducing host-derived damage-associated molecular patterns (DAMPs). However, like most other DAMP-inducing adjuvants such as aluminum hydroxide (Alum), HP-β-CyD may not be sufficient for the induction of protective type-1 (cellular) immune responses, thereby leaving room for improvement. Here, we demonstrate that a combination of HP-β-CyD with a humanized TLR9 agonist, K3 CpG-ODN, a potent pathogen-associated molecular pattern (PAMP), enhanced the protective efficacy of the co-administered influenza split vaccine by inducing antigen-specific type-2 and type-1 immune responses, respectively. Moreover, substantial antigen-specific IgE induction by HP-β-CyD, which can cause an allergic response to immunized antigen was completely suppressed by the addition of K3 CpG-ODN. Furthermore, HP-β-CyD- and K3 CpG-ODN-adjuvanted influenza split vaccination protected the mice against lethal challenge with high doses of heterologous influenza virus, which could not be protected against by single adjuvant vaccines. Further experiments using gene deficient mice revealed the unique immunological mechanism of action *in vivo*, where type-2 and type-1 immune responses enhanced by the combined adjuvants were dependent on TBK1 and TLR9, respectively, indicating their parallel signaling pathways. Finally, the analysis of immune responses in the draining lymph node suggested that HP-β-CyD promotes the uptake of K3 CpG-ODN by plasmacytoid dendritic cells and B cells, which may contributes to the activation of these cells and enhanced production of IgG2c. Taken together, the results above may offer potential clinical applications for the combination of DAMP-inducing adjuvant and PAMP adjuvant to improve vaccine immunogenicity and efficacy by enhancing both type-2 and type-1 immune responses in a parallel manner.

## Introduction

Adjuvants are used in vaccine formulations to induce potent immune responses that cannot be obtained with antigen alone (1). As the function of pattern recognition receptors (PRRs) such as Toll-like receptors (TLRs) became apparent over the last few decades, various PRR ligands have been developed for use as pathogen-associated molecular pattern (PAMP) adjuvants. For example, monophosphoryl lipid A and CpG-ODN are both used clinically as PAMP adjuvants (2, 3). In contrast, aluminum hydroxide (Alum), which is the most common adjuvant currently in clinical use, is not a PAMP (4). However, Alum stimulates the release of damage-associated molecular patterns (DAMPs) such as host DNA, which mediates its adjuvanticity (5). Therefore, not only PAMP adjuvants, but also DAMP-inducing adjuvants are worth developing.

Adjuvants are classified as either type-2 or type-1 adjuvants which enhance humoral and cellular immunity, respectively (6, 7). The most common adjuvant, Alum, induces type-2 immune responses and enhances vaccine efficacy (8, 9). Therefore, it has been empirically proven that adjuvant-induced type-2 immune responses are very effective in protecting against infectious diseases. In contrast, type-1 adjuvants such as poly I:C and CpG-ODN enhance cellular immunity, including the induction of Th1 cells and the activation of cytotoxic T lymphocytes, NK cells and phagocytes (10–14). Moreover, Th1 cells induce Th1-related immunoglobulins which also contributes to the elimination of pathogens (15–19). Thus, type-2 type and type-1 type immune responses have different functions in the defense against infection, and the development of an adjuvant capable of inducing both immune responses may be preferable.

Cyclodextrin (CyD) is a cyclic malto-oligosaccharide and includes α, β, and γ-CyD. CyDs encapsulate hydrophobic compounds and contribute to their solubilization and stabilization (20, 21). Moreover, biomedical applications of CyDs are attractive due to their high biocompatibility (22). Hence, they have been used as pharmaceutical additives (23–25). Moreover, applications of CyDs as active pharmaceutical ingredients (API) are also being developed for the treatment of various diseases (26–29). Notably, 2-hydroxypropyl-β-CyD (HP-β-CyD) has already been administered in a clinical trial to suppress the neurological symptoms in Niemann-Pick type C disease patients (30). Accordingly, repositioning of HP-β-CyD as a novel API to treat other diseases or for use in therapeutic modalities is currently being explored.

Recently, Onishi et al. reported that subcutaneous injection of HP-β-CyD induces the release of DAMPs including host DNA and enhances antigen-specific humoral immunity, both of which are similar to the adjuvant effect of Alum (31). Furthermore, intranasal administration of HP-β-CyD-adjuvanted influenza split vaccine (SV), as well as via the more traditional subcutaneous route, significantly improved the survival rate from influenza viral infection (32). These reports suggest the possibility of using HP-β-CyD as a novel adjuvant with few side effects. However, like most other DAMP-inducing adjuvants such as Alum, HP-β-CyD alone may not be sufficient for induction of protective type-1 (cellular) immune responses, thereby leaving room for improvement (31).

Therefore, we investigated improving the DAMP-inducing adjuvant effect of HP-β-CyD by combining it with PAMPs that can enhance type-1 immune responses. We mainly focused on CpG oligodeoxynucleotide (ODN) which is short synthetic single-stranded DNA containing unmethylated CpG dinucleotides. CpG-ODN induces type-1 immune responses via the stimulation of TLR9 which is located in endosomes (33). K type CpG-ODN is generally available as a vaccine adjuvant for human use due to its suitable pharmaceutical properties (34). Notably, CpG-ODN has already been approved for clinical use as a hepatitis B virus vaccine adjuvant (3). Furthermore, some studies show that K type CpG-ODN synergistically induces a type-1 immune response when used in combination with other adjuvants (35, 36). Thus, we chose K3 CpG-ODN as a potent type-1 adjuvant candidate and examined whether the combination of HP-β-CyD and K3 CpG-ODN induces better, synergistic, parallel, or distinct immune responses compared with the single adjuvants, and validated their efficacy in a mouse model of influenza split vaccination challenged with a lethal dose of influenza virus.

## Materials and methods

### Mice

Wild-type (WT) C57BL/6 mice (female) were purchased from CLEA Japan, Inc. (Osaka, Japan). Mice deficient for *Tlr9, Tnf/Tbk1, Tmem173, Ips-1, Irf-3*, and *Il-12p40* (male and female) were generated as previously described (31, 35). In brief, *Tlr9*- and *Ips-1*-knockout mice were purchased from Oriental BioService (Kyoto, Japan). *Irf3*-knockout mice were provided by the RIKEN BioResourse Center (Ibaraki, Japan). *Il-12p40*-knockout mice were purchased from the Jackson Laboratory (Maine, U.S.A.). *Tmem173*-knockout mice were generated from *Tmem173*^*tm*1*Camb*^ (KOMP)Mbp ES cell line (JM8A3.N1) obtained from Knockout Mouse Project (KOMP) Repository (California, U.S.A.). *Tnf/Tbk1*-knockout mice were prepared as described (37). These mice were used for the experiments at 6–10 weeks of age. All animal procedures were carried out in accordance with the appropriate laws and with the approval of the Ethics Committee for Animal Research of Kumamoto University (Approval ID: A28-031) and the National Institutes of Biomedical Innovation, Health and Nutrition (DS22-34R20).

### Reagents

Ovalbumin (OVA) with low endotoxin content (Wako, Osaka, Japan) or monovalent influenza SV, containing influenza virus hemagglutinin (HA) surface antigen from New Caledonia/20/1999 (H1N1) (a kind gift from The Research Foundation for Microbial Disease of Osaka University) were used as antigens. HP-β-CyD (degree of substitution: 4.3) was kindly gifted by Nihon Shokuhin Kako (Tokyo, Japan). Non-labeled and Alexa Fluor^®;^ 594-labeled K3 CpG-ODN were synthesized by GeneDesign as previously described (38). Phosphate buffered saline (PBS), RPMI1640 medium, and penicillin/streptomycin were purchased from Nacalai Tesque (Kyoto, Japan).

### Immunization and culture of splenocytes

After anesthetization, mice were administered 3 μg of OVA in PBS (60 μL) containing 3, 10, or 30% (w/v) HP-β-CyD and K3 CpG-ODN (0.1, 1, or 10 μg) at the base of the tail on days 0 and 14. Sera were collected at 7 days after the boost injection. Then, the mice were euthanized and their spleens were collected. After hemolysis of red blood cells using ACK lysis buffer, splenocytes were cultured at a density of 1 × 10^7^ cells/mL in RPMI medium containing penicillin (100 units/mL)/streptomycin (100 μg/mL) and 10% fetal bovine serum, and stimulated with OVA (10 μg/mL) for 48 h. The concentrations of mouse IL-4, IL-5, and IFN-γ were measured using ELISA MAX™ sets in accordance with the manufacturer's instructions (Biolegend, San Diego, CA, USA).

### Measurement of antigen-specific antibodies

Titers of antigen-specific total IgG, IgG1, and IgG2c in serum were determined by ELISA as described previously (32). In brief, 96 well half-area microplate (Corning Inc., Corning, NY, USA) was coated with 10 μg/mL OVA or 1 μg/mL SV solution overnight at 4°C. After washing three times with PBS containing 0.05% Tween 20 (PBS-T), the plate was incubated with 1% bovine serum albumin in PBS for 1h at room temperature. Then, diluted serum was added to the wells. After 2 h, the plate was washed with PBS-T and incubated with horseradish peroxidase-conjugated anti-mouse IgG, IgG1, or IgG2c antibody (Southern Biotech, Birmingham, AL, USA) for 1 h. After washing with PBS-T, TMB Microwell Peroxidase Substrate System (KPL, Gaithersburg, MD, USA) was added to the well and the reaction was stopped with 2 N H_2_SO_4_ 20 min later. Titers of antigen-specific antibodies were determined by log-linear interpolation of the serum dilution value corresponding to cut-off absorbance (OD450 of 0.2). The concentration of anti-OVA IgE was determined using a DS Mouse IgE ELISA (OVA) kit (DS Pharma Biomedical Co., Ltd., Osaka, Japan).

### Efficacy of HP-β-CyD/K3 CpG-ODN-adjuvanted influenza SV in virus infection model

Mice were immunized at the base of the tail with 3 μg of influenza SV (in 60 μL PBS) containing 30% HP-β-CyD and 10 μg of K3 CpG-ODN on day 0 and 14. On day 21, peripheral blood was collected, then the mice were intranasally challenged with 50 or 200 LD_50_ of clinically isolated A/Puerto Rico/8/1934 influenza (H1N1) virus in 30 μL of PBS. Body weight and survival rate of the mice were monitored for 2 weeks post-infection. In addition to mice that were found dead, mice with a weight loss of more than 30% of the starting body weight were euthanized and recorded as dead.

### Evaluation of immune responses in the draining lymph node

Mice were administered 3 μg of OVA in PBS containing 30% HP-β-CyD and 10 μg of K3 CpG-ODN at the base of the tail. After 24 h, the draining lymph node was collected, and the weight and number of cells were measured. Then, the cells were incubated with anti-mouse CD16/32 antibody (clone 93; Biolegend) to block of Fc receptors and stained with the following antibodies from Biolegend: anti-mouse CD11c (clone N418), Siglec-H (clone 551), CD19 (clone 6D5), CD40 (clone 3/23), CD69 (clone FN50), CD86 (clone GL-1), and DEC205 (clone NLDC-145). Dead cells were detected using 7-AAD (eBioscience, San Diego, CA, USA) or Live/Dead™ fixable blue dead cell stain kit (Invitrogen, Life Technologies, Carlsbad, CA, USA) and excluded from the analysis. Data were obtained with a BD Accuri C6 or BD LSR II flow cytometer (BD Bioscience, San Jose, CA, USA) and analyzed by BD Accuri C6 software (BD Bioscience) or FlowJo software (Tree Star, Ashland, OR, USA).

### Evaluation of the uptake of K3 CpG-ODN

Mice were administered 3 μg of OVA in PBS containing 30% HP-β-CyD and 10 μg of Alexa Fluor^®;^594-labeled K3 CpG-ODN at the base of the tail. After 24 h, the draining lymph node was collected, and the cells were stained and measured following the procedure above. The percentages of Alexa Fluor^®;^594^+^ cells of conventional dendritic cells (CD11c^+^ Siglec-H^−^), plasmacytoid dendritic cells (CD11c^+^ Siglec-H^+^) and B cells (CD19^+^) were analyzed by FlowJo software (Tree Star).

### Statistics

All experiments were independently performed two or three times. The experimental results are shown as the means ± SEM. Statistical significance of the differences between groups was determined by an unpaired Student's *t*-test or one-way ANOVA with Bonferroni's multiple comparison test. The survival curves after viral infection were compared by a Kaplan-Meier analysis (log-rank test and Wilcoxon's test) with Bonferroni's multiple comparison test. A *P*-value less than 0.05 indicated a statistically significant difference.

## Results

### Combination of HP-β-CyD and K3 CpG-ODN induces both type-2 and type-1 immune responses while suppressing IgE induction

**Figure 1:**
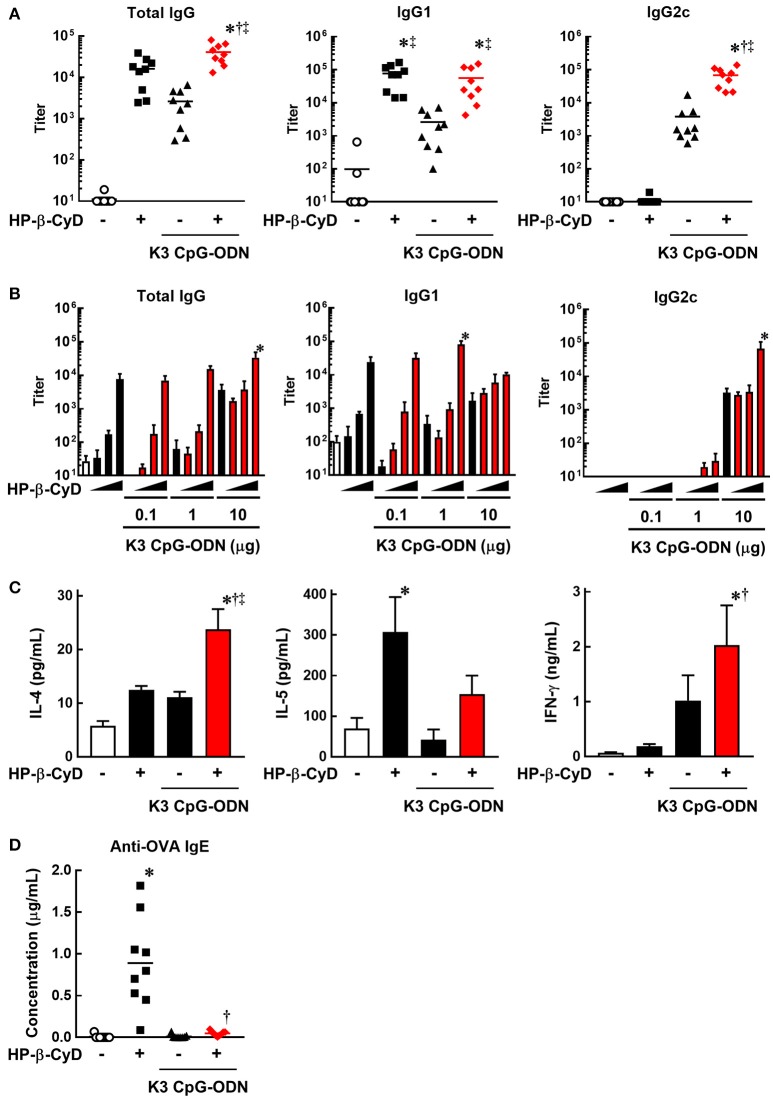
Combination of HP-β-CyD and K3 CpG-ODN induces both type-1 and type-2 responses in parallel with suppressed production of IgE. **(A–D)** C57BL/6 mice were inoculated with 3 μg of OVA in PBS containing **(A)** 30% HP-β-CyD and 10 μg of K3 CpG-ODN or **(B)** 3, 10, or 30% HP-β-CyD and K3 CpG-ODN (0.1, 1, or 10 μg) into the base of the tail on day 0 and 14. Blood was collected 7 days after the boost. Anti-OVA total IgG, IgG1, IgG2c **(A)** and IgE **(C)** were determined by ELISA. **(A,C)** Each dot represents an individual mouse (*n* = 8–9 per group in 2 independent experiments). **(B)** Each value represents the mean ± S.E. (*n* = 3–4 per group, representative data of two independent experiments). **(C)** Seven days after the second immunization, splenocytes were collected and cultured under stimulation with OVA (10 μg/mL). After 48 h, the concentrations of IL-4, IL-5, and IFN-γ in the medium were measured by ELISA. Each value represents the mean ± S.E. (*n* = 6 per group in two independent experiments). **(A–D)** *p < 0.05 compared with OVA alone,^†^*p* < 0.05 compared with OVA+HP-β-CyD, ‡*p* < 0.05 compared with OVA+K3 CpG-ODN (one-way ANOVA with Bonferroni's multiple comparison test).

To evaluate the induction of type-2 and type-1 immune responses by the combination of HP-β-CyD and K3 CpG-ODN, we immunized C57BL/6 mice with OVA solution containing these adjuvants at the base of the tail on days 0 and 14. At day 21, peripheral blood was collected, and OVA-specific antibodies were measured by ELISA. HP-β-CyD or K3 CpG-ODN alone induced the production of antigen-specific IgG1 or IgG2c which are indicators of type-2 and type-1 immune responses, respectively (Figure 1A). Importantly, this combination enhanced the production of both IgG1 and IgG2c. Notably, IgG2c production induced by the combined adjuvants was markedly higher than that of K3 CpG-ODN alone, while that of IgG1 was similar with HP-β-CyD alone (Figure 1A). We simultaneously evaluated optimal dose requirement for both adjuvants. The combination of 30% HP-β-CyD and 10 μg of K3 CpG-ODN elicited the highest total IgG and IgG2c titers (Figure 1B), so we performed the following experiments with this condition. Next, we evaluated T cell responses by measuring the cytokine production of splenocytes from the immunized mice. The combined adjuvant promoted the antigen-specific production of both IL-4 and IFN-γ, which are type-2 and type-1 cytokines, respectively (Figure 1C). These results suggest that the combination of HP-β-CyD and K3 CpG-ODN induces both type-2 and type-1 immune responses, and particularly enhances type-1 response cooperatively.

We then assessed IgE production, which is an unwanted or unnecessary Ig isotype as it can cause allergic response to immunized antigens. Type-2 adjuvants such as Alum often induce the production of IgE against the immunized antigens. However, the production of antigen-specific IgE induced by HP-β-CyD is significantly lower than that induced by Alum (31). Furthermore, it is known that K3 CpG-ODN suppresses the induction of IgE (39, 40). Consistent with these reports, the production of antigen-specific IgE induced by HP-β-CyD was completely suppressed by the addition of K3 CpG-ODN (Figure 1D). Therefore, the combination of K3 CpG-ODN contributes not only to the induction of type-1 immune response but also the improvement of the safety of HP-β-CyD administration.

### HP-β-CyD and K3 CpG-ODN cooperatively improve the efficacy of influenza SV against heterologous influenza virus infection in mice

Previously, we revealed that HP-β-CyD-adjuvanted influenza SV protected against a lethal dose of influenza virus (31, 32). Another type-2 adjuvant, Alum, is also an effective adjuvant for the influenza SV vaccine (41). In contrast, previous studies indicated that antibody-mediated responses such as antibody-dependent cell-mediated cytotoxicity (ADCC) and complement-dependent cytotoxicity (CDC) via Th1-related antibodies are also important for the elimination of influenza virus (16–19). Indeed, CpG-ODN is reported to enhance vaccine-induced type-1 (Th1) immune responses and protect the mice from lethal viral infections such as influenza (42, 43). Thus, the combination of type-2 and type-1 adjuvants is considered to cooperatively improve vaccine efficacy.

**Figure 2:**
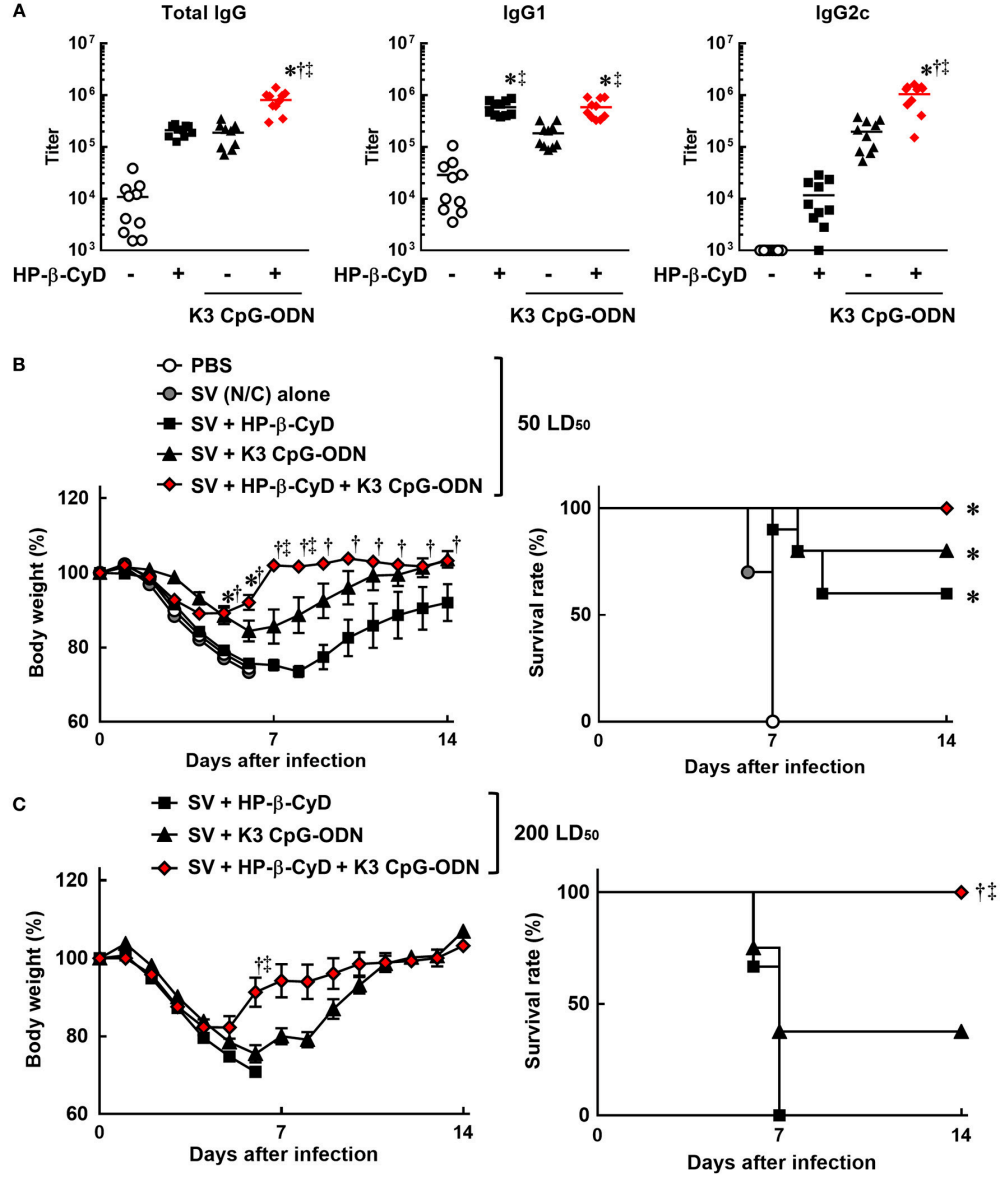
HP-β-CyD and K3 CpG-ODN cooperatively improve the efficacy of influenza split vaccine (SV) against influenza viral infection in mice. **(A–C)** C57BL/6 mice were inoculated with 3 μg of SV (New Caledonia strain) containing 30% HP-β-CyD and 10 μg of CpG-ODN into the base of the tail on day 0 and 14. **(A)** Blood was collected 7 days after boost and anti-HA total IgG, IgG1, and IgG2c were determined by ELISA. Each dot represents an individual mouse (*n* = 10 per group, representative data of 2 independent experiments). ^*^p < 0.05 compared with SV alone,^†^*p* < 0.05 compared with SV+HP-β-CyD,^‡^*p* < 0.05 compared with SV+K3 CpG-ODN (one-way ANOVA with Bonferroni's multiple comparison test). **(B,C)** Seven days after the last immunization, mice were challenged with 50 **(B)** or 200 **(C)** LD_50_ of influenza virus A/PR/8(H1N1) by intranasal administration and body weight and survival rate were monitored. Each point represents the mean ± S.E. (**B**; *n* = 4–10, **C**; *n* = 9 per group, representative data of 2 independent experiments). ^*^p < 0.05 compared with SV alone,^†^*p* < 0.05 compared with SV+HP-β-CyD,^‡^*p* < 0.05 compared with SV+K3 CpG-ODN [one-way ANOVA with Bonferroni's multiple comparison test for body weights or Kaplan-Meier analysis (log-rank test and Wilcoxon's test for survival curves comparisons)].

Therefore, we evaluated the efficacy of the combination of HP-β-CyD and K3 CpG-ODN as an adjuvant for influenza SV. Mice were injected with HP-β-CyD/K3 CpG-ODN-adjuvanted influenza SV (New Caledonia/20/1999 strain) at the base of the tail twice. The production of HA-specific total IgG, IgG1, and IgG2c after the second immunization was significantly increased by the addition of these adjuvants (Figure 2A). Furthermore, the combined adjuvants cooperatively enhanced the production of HA-specific IgG2c as with the case of OVA-specific responses (Figure 2A). Next, mice were intranasally challenged with a 50 LD_50_ dose of heterologous influenza virus A/Puerto Rico/8/1934 strain 1 week after the boost injection. HP-β-CyD/K3 CpG-ODN-adjuvanted influenza SV significantly improved both the body weight loss and survival rate compared with SV alone (Figure 2B). In contrast, more than half of the mice also survived after single adjuvant vaccines, which suggests that HP-β-CyD or K3 CpG-ODN alone can provide adequate immune response in this setting. Therefore, we performed this experiment with a higher dose of influenza virus (200 LD_50_). In this severe viral infection model, although the survival rate and body weight were decreased in the mice with single adjuvant vaccines, the mice immunized with the combination adjuvant showed more rapid weight recovery and 100% survival rate (Figure 2C). Taken together, these results suggest that the combination of HP-β-CyD and K3 CpG-ODN greatly improves the efficacy of influenza SV vaccine by inducing protective humoral responses that were likely benefited from the induction of protective cellular responses, which contribute to better host protection than their singular use.

### Induction of type-2 immune responses by HP-β-CyD and K3 CpG-ODN is dependent on TBK1

**Figure 3:**
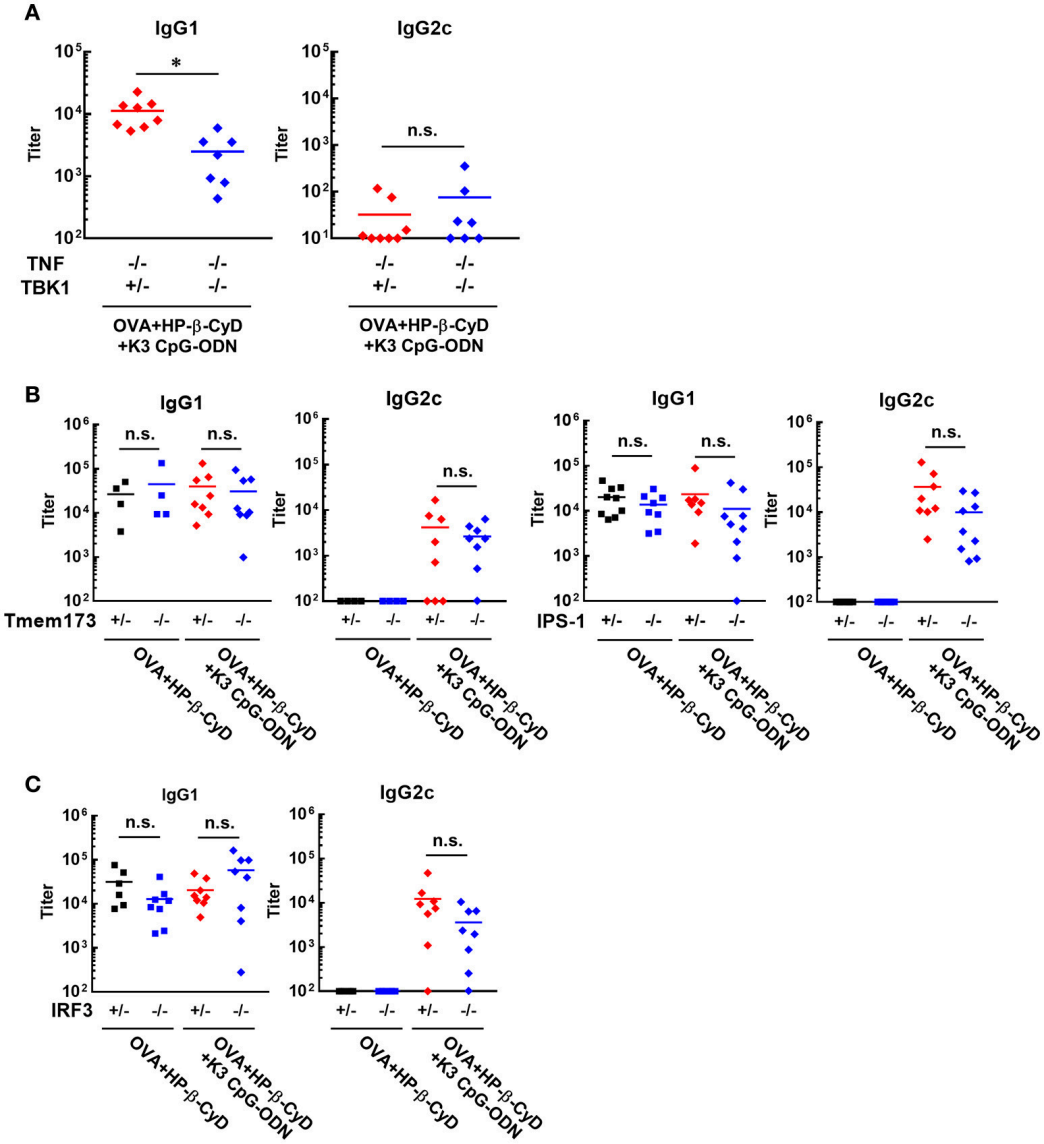
Induction of type-2 immune responses by HP-β-CyD and K3 CpG-ODN is dependent on TBK1. **(A–C)** C57BL/6 mice (**A**; *Tnf* ^−/−^*Tbk1*^+/−^ or *Tnf* ^−/−^*Tbk1*^−/−^, **B**; *Tmem173*^+/−^ or *Tmem173*^−/−^, *Ips-1*^+/−^ or *Ips-1*^−/−^, **C**; *Irf3*^+/−^ or *Irf3*^−/−^) were inoculated with 3 μg of OVA solution containing 30% HP-β-CyD and 10 μg of K3 CpG-ODN into the base of the tail on day 0 and 14. Blood was collected 7 days after the boost. Anti-OVA IgG1 and IgG2c were determined by ELISA. Each dot represents an individual mouse (**A**; *n* = 7–8, **B**; *n* = 4–9, **C**; *n* = 6–8 per group in two independent experiments, blue; gene deficient mice). ^*^p < 0.05, n.s., not significant (Student's *t*-test or one-way ANOVA with Bonferroni's multiple comparison test).

It is important to reveal the mode of action of adjuvants to prove their safety profile based on scientific evidence prior to future clinical application. Therefore, we investigated the mechanism of the adjuvanticity of HP-β-CyD and K3 CpG-ODN. Previously, Onishi M. et al. and Kusakabe T. et al. reported that HP-β-CyD induces temporal release of host DNA after subcutaneous and intranasal administration, and its adjuvanticity is mediated by TANK-binding kinase 1 (TBK1) (31, 32). Thus, we used *Tnf* ^−/−^*Tbk*1^−/−^ mice (the deficiency of TBK1 leads the death *in utero*, and this lethal effect can be reduced in the absence of TNF (37)) to examine whether the combination of HP-β-CyD and K3 CpG-ODN also enhances type-2 immune responses via TBK1. The production of antigen-specific IgG1 was partially decreased by the knockout of TBK1 (Figure 3A), which corresponds with previous results of singular use of HP-β-CyD (31). It was difficult to assess whether TBK1 also contributes to type-1 immune responses induced by the combined adjuvant in this experiment because significant production of antigen-specific IgG2c was not determined either TBK1 hetero or KO mice, probably due to the deficiency of TNF.

TBK1 is activated by several upstream adaptors such as STING and IPS-1. Therefore, we next examined which signaling cascade is involved in TBK1-mediated type-2 immune responses using *IPS-1*^−/−^ and *Tmem173*^−/−^ (STING KO) mice. However, the deficiency of these adaptors did not affect the adjuvanticity of HP-β-CyD alone or combined adjuvant (Figure 3B). Furthermore, the knockout of IRF3, which is downstream of TBK1, did not affect the adjuvanticity (Figure 3C). Thus, although the detailed mechanism is still unclear, these results suggest that type-2 immune responses induced by HP-β-CyD and K3 CpG-ODN are partially dependent on TBK1.

### Induction of type-1 immune responses by HP-β-CyD and K3 CpG-ODN is dependent on TLR9-mediated signaling and the enhanced production of IL-12

**Figure 4:**
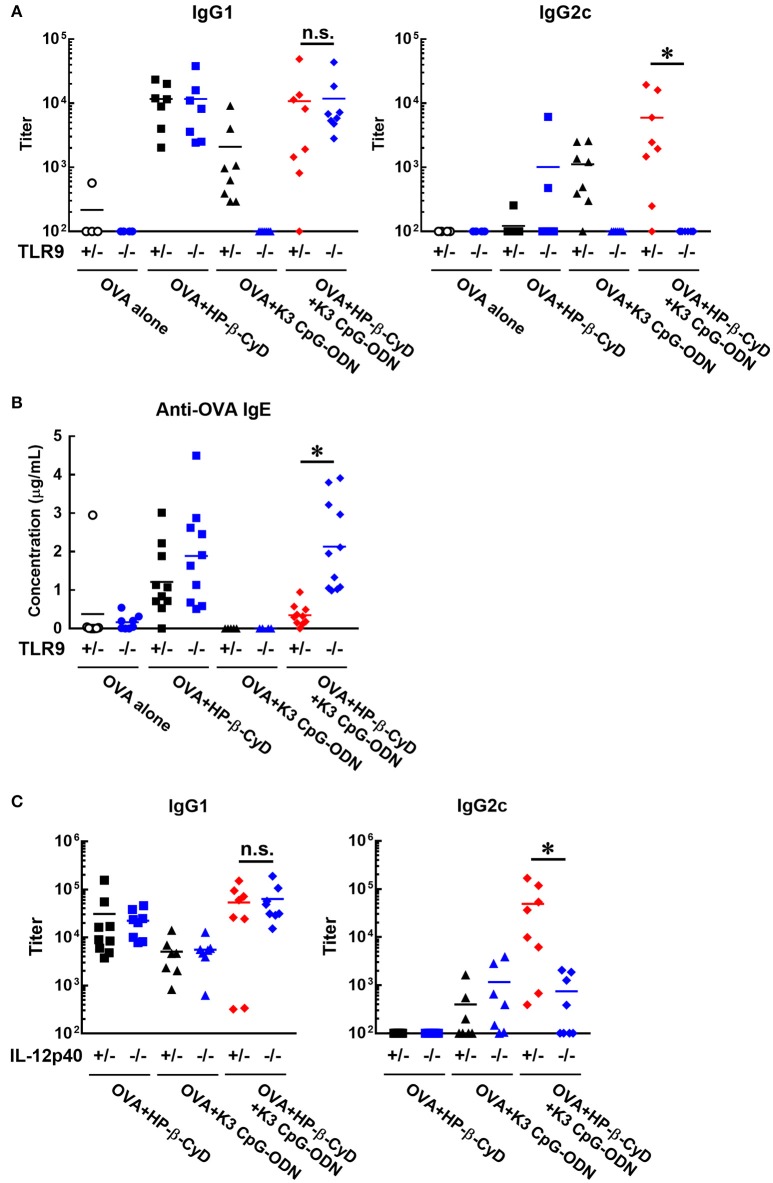
Induction of type-1 immune responses by HP-β-CyD and K3 CpG-ODN is dependent on TLR9-mediated signaling and the production of IL-12p40. **(A–C)** C57BL/6 mice (**A,B**; *Tlr9*^+/−^ or *Tlr9*^−/−^, **C**; *Il-12p40*^+/−^ or *Il-12p40*^−/−^) were inoculated with 3 μg of OVA in solution containing 30% HP-β-CyD and 10 μg of K3 CpG-ODN into the base of the tail on day 0 and 14. Blood was collected 7 days after the boost. Anti-OVA IgG1, IgG2c and IgE were determined by ELISA. Each dot represents an individual mouse (**A**; *n* = 4–8, **B**; *n* = 4–11, **C**; *n* = 7–9 per group in two independent experiments, blue; gene deficient mice). ^*^p < 0.05 (one-way ANOVA with Bonferroni's multiple comparison test).

Next, we investigated the effect of TLR9 deficiency on the adjuvanticity of the combination of HP-β-CyD and K3 CpG-ODN because CpG-ODN induces immune responses via activation of TLR9 (33). Consistent with a previous report, the induction of antigen-specific IgG1 and IgG2c by K3 CpG-ODN alone was completely suppressed in *Tlr9*^−/−^ mice. In contrast, the deficiency of TLR9 inhibited the production of IgG2c but not IgG1 in the combination adjuvant-treated group (Figure 4A). These results suggested that TLR9-mediated signaling is essential for type-1 immune response induced by combination adjuvant, and the induction of type-1 and type-2 immune responses is mediated by different signals. We also measured antigen-specific IgE in these mice. The production of IgE by the combined adjuvant was increased by the knockout of TLR9 (Figure 4B). Thus, the suppressive effect of K3 CpG-ODN on IgE production induced by HP-β-CyD was dependent on TLR9-mediated signaling.

To reveal a more detailed mechanism of the induction of type-1 immune responses, we used *Il-12p40*^−/−^ mice as IL-12 is a major type-1 cytokine. Notably, the production of IgG2c, but not IgG1, induced by the combined adjuvant was significantly decreased by the knockout of IL-12p40 (Figure 4C). From these results, type-1 responses induced by HP-β-CyD and K3 CpG-ODN were completely dependent on TLR9-mediated signaling and enhanced production of IL-12.

### HP-β-CyD and k3 CpG-ODN cooperatively stimulate immune response in the draining lymph node

**Figure 5:**
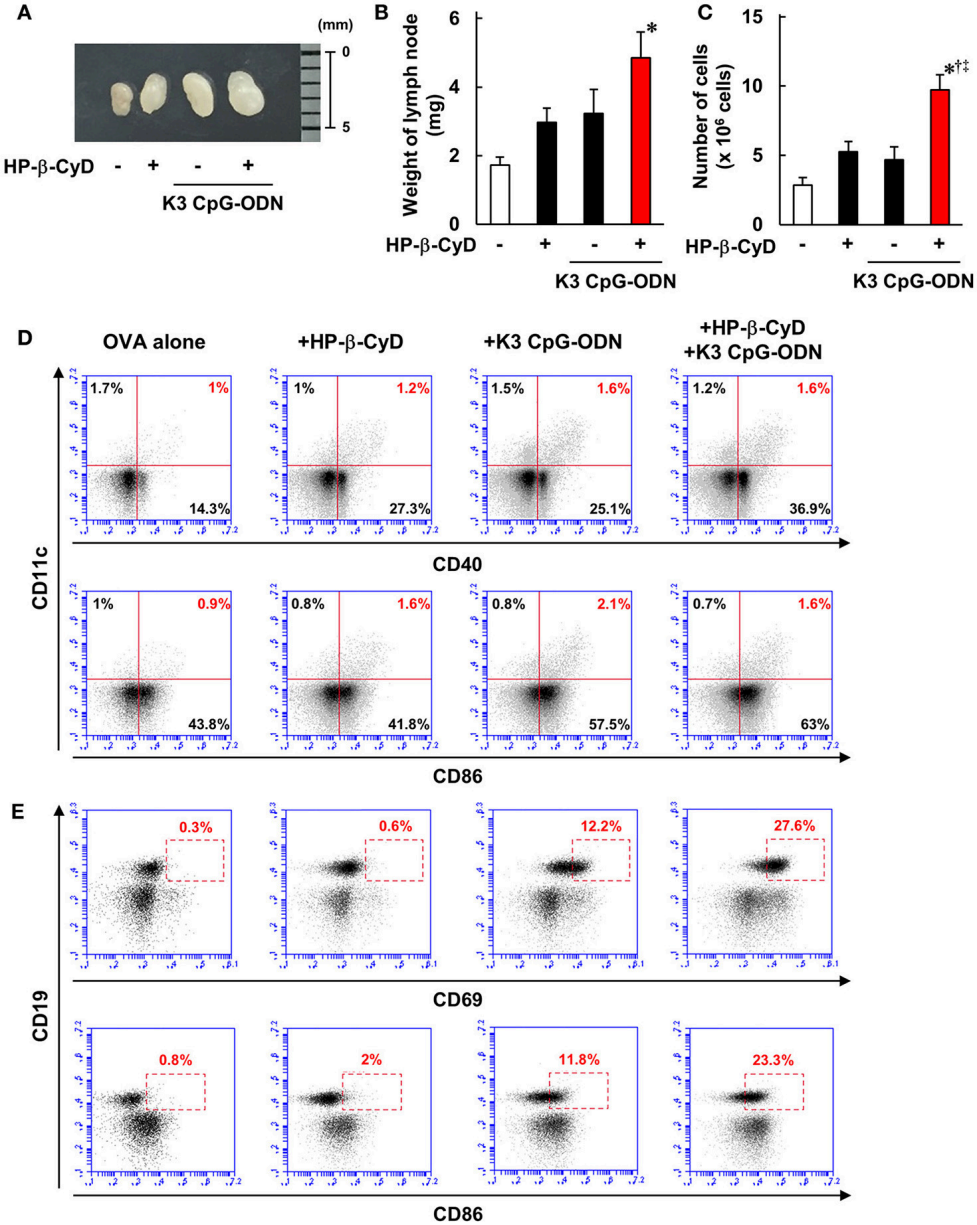
HP-β-CyD and K3 CpG-ODN cooperatively enhance the immune response in the draining lymph node. **(A–E)** C57BL/6 mice were inoculated with 3 μg of OVA solution containing 30% HP-β-CyD and 10 μg of K3 CpG-ODN into the base of the tail. The draining lymph node was collected after 24 h. After taking a photograph, the weight of lymph node and the number of lymphocytes was measured. **(B,C)** Each value represents the mean ± S.E. (*n* = 6 per group in two independent experiments). ^*^p < 0.05 compared with OVA alone,^†^*p* < 0.05 compared with OVA+HP-b-CyD,^‡^*p* < 0.05 compared with OVA+K3 CpG-ODN (one-way ANOVA with Bonferroni's multiple comparison test). **(D,E)** The expression of CD40, CD69, and CD86 on CD11c^+^ or CD19^+^ cells was analyzed by flow cytometry. The experiments were performed independently three times, and representative data are shown.

Knockout mouse studies suggest that the induction of type-1 responses by the combined adjuvant was completely dependent on TLR9. In contrast, although HP-β-CyD enhances immune responses without the requirement of TLR9 [Figure 4A, (31)], type-1 immune responses by K3 CpG-ODN were enhanced in the presence of HP-β-CyD (Figure 1A), which led us to hypothesize that HP-β-CyD plays a supportive role in the induction of type-1 responses. To elucidate this possibility, we examined the early immune responses after the administration of HP-β-CyD and K3 CpG-ODN. First, we collected the draining lymph node 24 h after vaccine injection and measured its weight and the number of cells. HP-β-CyD and K3 CpG-ODN cooperatively promoted the expansion of draining lymph node (Figures 5A–C). Then, we analyzed the expression of activation markers (CD40, CD69, and CD86) on immune cells by using flow cytometry. It is known that dendritic cells (DCs) and B cells highly express TLR9 (44, 45), therefore we focused on the activation of these cells. HP-β-CyD and K3 CpG-ODN increased the frequency of activated DCs (CD11c^+^ cells) and B cells (CD19^+^ cells) (Figures 5D,E). Interestingly, HP-β-CyD itself did not affect B cells, but the combined adjuvant significantly enhanced the activation of B cells compared with K3 CpG-ODN alone. We also performed the experiment at 48 h later, but activated DCs and B cells tended to decrease, suggesting that the enhancement of immune responses in the draining lymph node by the these adjuvants was maximized within first 24 h (Supplementary Figure 1). These results suggest that HP-β-CyD and K3 CpG-ODN stimulate immune cells including DCs, and synergistically activated B cells in the early phase.

### HP-β-CyD enhances the uptake of K3 CpG-ODN by plasmacytoid DCs and B cells

**Figure 6:**
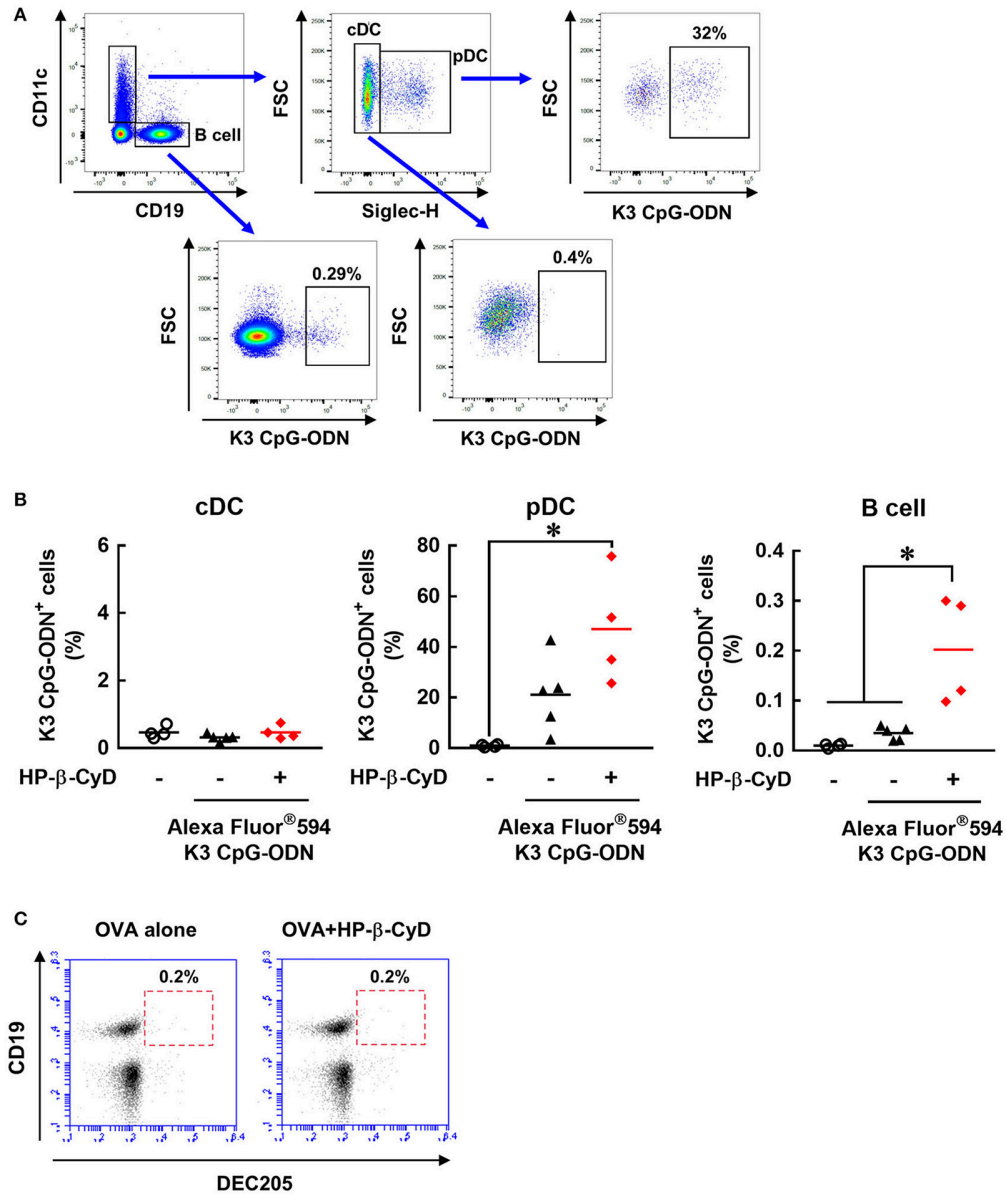
HP-β-CyD enhances the uptake of K3 CpG-ODN by pDCs and B cells in the draining lymph node. **(A,B)** C57BL/6 mice were injected with OVA solution containing 30% HP-β-CyD and 10 μg of Alexa Fluor^®;^594-labeled K3 CpG-ODN into the base of the tail. After 24 h, the draining lymph node was collected, and the uptake of K3 CpG-ODN by CD11c^+^ Siglec-H^−^ cells (cDC), CD11c^+^ Siglec-H^+^ cells (pDC) and CD19^+^ cells (B cell) was analyzed by flow cytometry. **(A)** Gating strategy after the exclusion of doublets and dead fluorescent positive cells was shown. **(B)** Each dot represents an individual mouse (*n* = 4 per group in 2 independent experiments). ^*^p < 0.05 (one-way ANOVA with Bonferroni's multiple comparison test). **(C)** C57BL/6 mice were inoculated OVA solution containing 30% HP-β-CyD into the base of the tail. After 24 h, draining lymph node was collected, and the expression of DEC205 on CD19^+^ cells was analyzed by flow cytometry. The experiments were performed independently twice, and representative data are shown.

We attempted to further investigate how HP-β-CyD and K3 CpG-ODN cooperatively activate DCs and B cells. Because the stimulation by K3 CpG-ODN seems to be essential, especially for innate activation of B cell (Figure 5E), we hypothesized that HP-β-CyD contributes to the uptake of K3 CpG-ODN by these cells. To examine this possibility, we administered fluorescently-labeled K3 CpG-ODN with or without HP-β-CyD, and the frequency of the K3 CpG-ODN positive cells was analyzed by flow cytometry (Figure 6A). Conventional DCs (cDCs) (CD11c^+^ Siglec-H^−^ cells) did not internalize K3 CpG-ODN. In contrast, some plasmacytoid DCs (pDCs) (CD11c^+^ Siglec-H^+^ cells) and B cells (CD19^+^ cells) demonstrated uptake of K3 CpG-ODN, and the proportion of fluorescent positive B cells were increased by the addition of HP-β-CyD (Figure 6B). The uptake of K3 CpG-ODN in pDC was also enhanced although it was not significant (*p* = 0.0693). Therefore, it was suggested that HP-β-CyD promotes the uptake of K3 CpG-ODN, which may contribute to the cooperative activation of immune cells. However, how HP-β-CyD enhanced the internalization of K3 CpG-ODN was unclear. It has been reported that CpG-ODN is internalized by the cell surface receptor DEC205 (46). Hence, we examined whether HP-β-CyD upregulates the expression of DEC205. Interestingly, its expression level was not affected by the administration of HP-β-CyD (Figure 6C). Thus, although the detailed mechanism of enhanced uptake of K3 CpG-ODN by HP-β-CyD remains unknown, it may contribute to the activation of DCs and B cells and the increase of type-1 immune response.

## Discussion

In this study, we evaluated the cooperative adjuvant effect of the combination of HP-β-CyD and K3 CpG-ODN as DAMP-inducing and PAMP adjuvants, respectively. This combination induced both type-2 and type-1 immune responses in parallel, particularly increasing type-1 immune responses without the production of IgE and cooperatively contributing to the efficacy of influenza SV.

Development of an adjuvant capable of inducing both type-2 and type-1 immune responses may be beneficial in certain applications that require both humoral and cellular immune responses for optimal efficacy. However, the most commonly available DAMP-inducing adjuvant, Alum, can induce humoral responses, but induces only negligible cellular immune responses. In contrast, PAMP adjuvants, like most TLR agonists, are good at inducing cellular immune responses. Many studies investigating the combination of Alum and DAMP-inducing adjuvants have been carried out to enhance type-1 immune response as well as type-2 immune response. For instance, AS04, which is composed of Alum and monophosphoryl lipid A, was clinically approved as a potent inducer of both type-2 and type-1 immune responses (47, 48). Thus, the combination of DAMP-inducing and PAMP adjuvants appears to be preferable to induce adequate immune responses which cannot be obtained by their singular use. However, combined adjuvants sometimes inhibit each other's adjuvanticity because Th2 and Th1 cells generally inhibit each other's function. Notably, CpG-ODN is known to suppress Th2 responses instead of solely the enhancement of Th1 response (49, 50). Thus, a study investigating novel combined adjuvants is desired for better understanding of immune responses after the co-administration of DAMP-inducing and PAMP adjuvants. The combination of HP-β-CyD and K3 CpG-ODN enhanced both type-2 and type-1 responses without inhibiting either response (Figure 1A). This may be attributable to the separate regulation of these responses.

Our result suggested that type-2 immune responses induced by the combined adjuvant were mediated by a TLR9-independent and partially TBK1-dependent mechanism, the same as the adjuvanticity of HP-β-CyD alone (Figures 3A, 4A). Moreover, the production of IgG1 by the combined adjuvant was equivalent to that of HP-β-CyD alone. Thus, these results suggest that K3 CpG-ODN was not essential for the combined induction of IgG1. Moreover, the production of IgG1 by HP-β-CyD or the combination adjuvant was not affected by the knockout of IPS-1 or STING (Figure 3B) and IRF3 (Figure 3C), which are well known upstream and downstream adaptors of TBK1, suggesting that other TBK1-mediated signaling cascades promotes the production of IgG1. Recently, it was revealed that TBK1 is activated by the inducible T cell co-stimulator (ICOS) in T follicular B helper cells, which promote the formation of germinal centers (GC) and the production of antigen-specific antibody although the detailed mechanism remains unknown (51). Thus, HP-β-CyD and combination adjuvants may enhance such TBK1-mediated immune response via the release of DAMPs. Notably, the suppressive effect of the knockout of TBK1 on IgG1 production was partial, not complete, so a TBK1-independent mechanism also seems to be involved in the induction of type-2 immune response. In contrast, the induction of IgE production by HP-β-CyD was completely suppressed by the combination of K3 CpG-ODN (Figure 1C). These data may imply that the production of IgG1 and IgE induced by HP-β-CyD is regulated by different mechanisms. As another DAMP-inducing adjuvant, Alum was reported to enhance the production of IgG1 through IRF3-independent signaling, while IgE production was in a IRF3-dependent manner, these different signaling pathways may be associated with the generation of Tfh2 cells and Th2 cells (5). The mechanism of IgG1 and IgE production by HP-β-CyD seems to be similar to that of Alum because the knockout of IRF3 did not affect the IgG1 response induced by HP-β-CyD, with or without K3 CpG-ODN (Figure 3C). Thus, there is a possibility that K3 CpG-ODN negatively affected only IRF3-mediated signaling which induces IgE production. Another factor to explain the separate regulation of IgG1 and IgE is the structural difference of the class-switching in B cells. It is reported that both IgG1^+^ and IgE^+^ B cells differentiate into plasma cells via affinity maturation in GC, although IgE^+^ B cells tend to be located outside the GC (52). Hence, it may be possible that K3 CpG-ODN could access B cells outside of the GC and suppress their differentiation into IgE-producing cells. Although the detailed mechanism should be investigated to account for the different regulation of IgG1 and IgE production, our results strongly suggest that combination of HP-β-CyD and K3 CpG-ODN may contribute to the understanding of type-2 immune responses.

The production of IgG2c by the combined adjuvant was significantly higher than that of K3 CpG-ODN alone, while HP-β-CyD alone did not induce IgG2c production. Moreover, the induction of type-1 immune responses was completely dependent on TLR9-mediated signaling. Thus, TBK1-dependent or independent signaling driven by HP-β-CyD may play a supportive role in the enhancement of TLR9-mediated type-1 immune responses. Unfortunately, we could not reveal whether TBK1-mediated signaling contributes to the induction of type-1 immune responses by the combined adjuvant because the production of IgG2c was not detected in either *TBK1*^+/−^ or *TBK1*^−/−^ mice, probably due to the deficiency of TNF, which is known to enhance type-1 immune responses (53). However, some studies show that TBK1-mediated signaling is involved in the induction of Tfh cells (51, 54, 55). Thus, HP-β-CyD might promote the induction of Th1-like Tfh cells via the activation of TBK1 in the combination with K3 CpG-ODN. Other mechanisms may also affect the induction of type-1 immune responses because the immune reaction induced by HP-β-CyD is likely to involve TBK1-independent mechanisms. To investigate one explanation regarding the enhanced type-1 response, we examined the immune response after the administration of HP-β-CyD and K3 CpG-ODN. This combination of adjuvants increased the expression of co-stimulatory molecules on dendritic cells and B cells, especially activating B cells (Figures 5D,E). Furthermore, the internalization of K3 CpG-ODN in pDCs and B cells was increased by co-administration of HP-β-CyD (Figure 6B). Thus, the increased type-1 immune response by the addition of HP-β-CyD may be due to the enhanced uptake of K3 CpG-ODN. Therefore, we analyzed the expression of DEC205, which is a cell surface receptor for CpG-ODN. However, HP-β-CyD did not affect its expression (Figure 6C), which suggests the existence of another mechanism. Previously, it was reported that the DAMP high-mobility group box 1 (HMGB1) interacts with CpG-ODN and accelerates its delivery to its receptor (56). Thus, the release of the DAMPs by the administration of HP-β-CyD might enhance the uptake of K3 CpG-ODN. The detailed mechanism of cooperative induction of the type-1 immune responses by combined adjuvants is an issue for future research.

We applied the combination of HP-β-CyD and K3 CpG-ODN to influenza SV. It is known that the efficacy of influenza vaccines in the elderly is lower than younger recipients. Infants also cannot obtain an adequate protective effect by the administration of vaccine antigen alone because most of them do not have memory cells against influenza virus due to the lack of previous influenza infection. Moreover, the risk of death by influenza infection is high in the elderly and infants. Therefore, the development of a safe and effective influenza vaccine is an urgent global issue. HP-β-CyD and K3 CpG-ODN-adjuvanted influenza SV protected against challenge with a high dose of influenza virus, which the single adjuvant vaccines could not protect against (Figure 2), suggesting that the anti-viral immune response generated by this combination of adjuvants is very strong. Notably, this adjuvant combination greatly induced IgG2c production, which is reported to be correlated with anti-viral immunity including ADCC and CDC (15–19). In contrast, the production of IgE by the combined adjuvant was lower than that of HP-β-CyD alone, and the biocompatibility of these adjuvants is expected to be high because they have already been used in the clinic. Taken together, the combination of HP-β-CyD and K3 CpG-ODN is a promising adjuvant for not only influenza SV but also other vaccines which require both type-1 and type-2 immune responses.

In conclusion, in the present study, we revealed that the combination of HP-β-CyD and K3 CpG-ODN is a novel adjuvant which induces both type-2 and enhanced type-1 responses without the production of IgE and the mechanism of action of the combination. These findings provide valuable information regarding the development of novel vaccines incorporating the combination of DAMP-inducing adjuvants and PAMP adjuvants.

## Author contributions

ToH, MM, TK, SK, KoM, YO, YS, FN, and ML performed the experiments. TaH, EK, MM, TK, KI, and HA analyzed the data. ToH, EK, MM, TK, CC, RO, TaH, KeM, KI, and HA designed the research. ToH, KI, and HA wrote the manuscript. KI and HA supervised this work.

### Conflict of interest statement

The authors declare that the research was conducted in the absence of any commercial or financial relationships that could be construed as a potential conflict of interest.
