# Supplementary material for: DAMP-Inducing Adjuvant and PAMP Adjuvants Parallelly Enhance Protective Type-2 and Type-1 Immune Responses to Influenza Split Vaccination
Source: Front Immunol. 2018 Nov 20;9:2619. doi: 10.3389/fimmu.2018.02619 (PMC6255964; doi:10.3389/fimmu.2018.02619)
Supplement: Supplementary file 1 [file Data_Sheet_1.docx]

Supplementary Material

**DAMP-inducing adjuvant and PAMP adjuvants parallelly enhance protective type-2 and type-1 immune responses to influenza split vaccination**

Tomoya Hayashi^1,2^, Masatoshi Momota^2,3^, Etsushi Kuroda^2,3^, Takato Kusakabe^2,3^, Shingo Kobari^2^, Kotaro Makasaka^1^, Yoshitaka Ohno^1, 4^, Yusuke Suzuki^1^, Fumika Nakagawa^1^, Michelle S. J. Lee^5^, Cevayir Coban^5^, Risako Onodera^6^, Taishi Higashi^1^, Keiichi Motoyama^1^, Ken J. Ishii^2,3,*^ and Hidetoshi Arima^1,4,*^

*** Correspondence:** Ken J. Ishii (kenishii@biken.osaka-u.ac.jp) and Hidetoshi Arima (arimah@gpo.kumamoto-u.ac.jp)


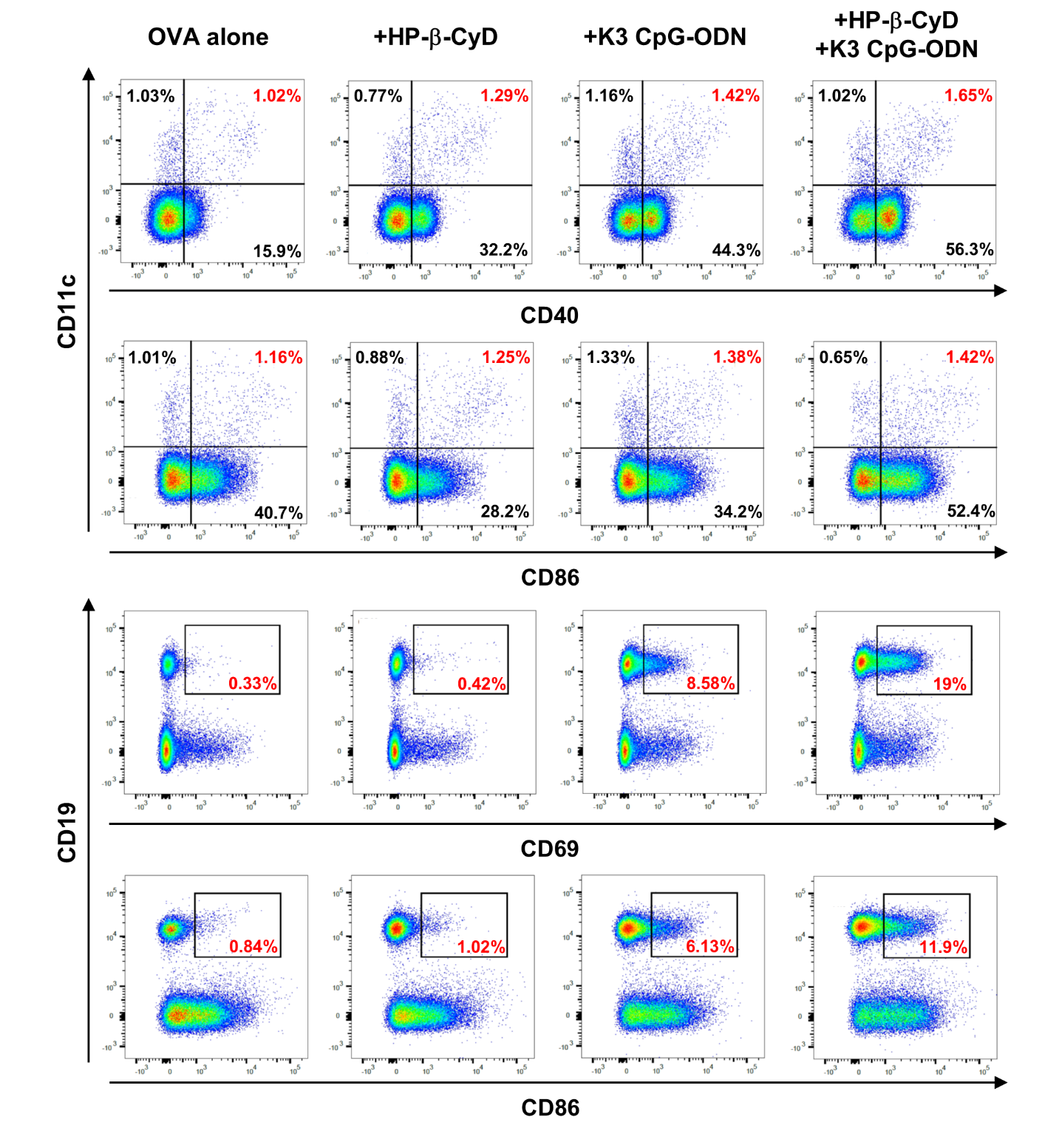


**Supplementary Figure 1.** Effect of HP-β-CyD and K3 CpG-ODN on the expression of co-stimulatory molecules on dendritic cells and B cells. C57BL/6 mice were inoculated with 3 μg of OVA solution containing 30% HP-β-CyD and 10 μg of K3 CpG-ODN into the base of the tail. The draining lymph node was collected after 48 h. The expression of CD40, CD69, and CD86 on CD11c^+^ or CD19^+^ cells were analyzed by flow cytometry. The experiments were performed independently twice, and representative data are shown.
